# Book Review: Corrupt Research: The Case for Reconceptualizing Empirical Management and Social Science

**DOI:** 10.3389/fpsyg.2016.01905

**Published:** 2016-12-05

**Authors:** Ashley E. Barnett

**Affiliations:** History and Philosophy of Science, The University of MelbourneMelbourne, VIC, Australia

**Keywords:** research credibility, null hypothesis significance testing, hypothetico-deductivism, replication crisis, philosophy of science

Raymond Hubbard documents the statistical, social, and philosophical causes of the “reproducibility crisis” in *Corrupt Research: The Case for Reconceptualizing Empirical Management and Social Science.* Hubbard's diagnosis of the reproducibility crisis includes a detailed critique of Null Hypothesis Significance Testing (NHST), in particular testing nil null hypotheses (hypotheses of “no difference”). His critique is supported by an almost exhaustive reference list, making *Corrupt Research* a useful text even if you are very familiar with the issue. Less familiar to readers may be his discussion of how the widespread tacit acceptance of a philosophically naive form of Hypothetico-Deductivism (HD) as *the* Scientific Method permits and exacerbates the other complementary causes. According to Hubbard, HD is erroneously thought to legitimize the inappropriate use of NHST and other methodological flaws.

The HD model in question prescribes that scientists first form a hypothesis, in some unspecific way, then deduce predictions of the hypothesis, and finally test those predictions. In these vague terms, the model does describe one important type of scientific reasoning, so if you ignore questions about the applicability of the HD method to many areas of psychology and how little the model tells you about the confirmatory value of experimental results, not to mention the scientists such as Darwin, Skinner, and many others who used very different inferences, HD does possess a prima facie plausibility. That along with its vague formulation, permits and seems to justify the use of NHST to apply HD by providing a method for calculating whether a hypothesis has been disconfirmed.

In turn, NHST's dominance has given the illusion that all scientific practice fits the HD model. In Hubbard's account, NHST and HD together cause a range of problems, such as the widespread practice of pretending that post-hoc hypothesizing happens *a priori*. Hubbard tells a compelling story regarding the proposed connection between HD and NHST, and he cites many corroborating critics, but his argument consists of circumstantial evidence, as he doesn't cite any authorities using HD to explicitly endorse NHST, or vice versa, or provide a survey of literature or textbooks showing that it is standardly presented as doing so. After his discussion of the beliefs and behaviors associated with HD his strongest conclusion is, “Given the above beliefs, it is understandable why tests of statistical significance have enjoyed such a privileged status in conventional social and business research methodology” (Hubbard, 2016, p. 38).

The connection between HD and NHST is one part of what Hubbard calls the Significant Difference approach to science. Hubbard thinks that the Significant Difference approach to science is widespread and causes great harm because it promotes an artificial and misleading view of how science should be done. This approach presents scientific discoveries as a series of neat and tidy questions that can be answered with precise and objective answers with NHST. Hypothesis discovery is neglected because testing is prioritized under the error that these are very separate activities. Descriptive correlation analysis is emphasized over the search for causal explanations. Overgeneralizing is common within the Significant Difference approach, because internal validity takes precedence over external validity, and answers to questions are given based on one data set without considerations of context. Looking at how NHST is typically presented by statisticians, Hubbard shows how scientists could be forgiven for thinking that it provides a conclusive and objective test of a scientific hypothesis, and hence contributes to the publication bias toward positive and novel results at the exclusion of replications. Hubbard provides concerning examples of the over-reliance on single studies, such as marketing textbooks teaching that subliminal messaging can increase sales, when this surprising finding was based on a single 1956 study that lacked scientific controls.

To the extent that the Significant Difference approach does consider replication it does does so insufficiently: “A replication success is defined as a result that was statistically significant in the initial study and continues to be so (in the same direction) in the follow up” (Hubbard, 2016, p. 71). But this ignores the low statistical power of most studies, which Hubbard rightly labels as “an empirical regularity not to be proud of” (Hubbard, 2016, p. 53). Hubbard presents a table of 16 studies that have examined the statistical power of articles from social science and psychology journals. For medium effect sizes there is only about a 60% chance of rejecting a false null hypothesis. “In short the significant difference paradigm legislates bad science” (Hubbard, 2016, p. 49).

There are many other reasons to read this book. It covers related sociological issues, such as the dangers of publish or perish culture, the evidence that peer review is “inclined to treat genuinely innovative, risk-taking, research harshly” (Hubbard, 2016, p.238), and the tension between knowledge advancement and career advancement. Particularly useful is Hubbard's point-by-point comparison of Significant Difference with his preferred approach called Significant Sameness. Hubbard argues that CIs should replace NHST, because CIs encourage the reader to “think meta-analytically about estimation, replication, and comparing intervals across studies” (Hubbard, 2016, p.70). Hubbard claims that critical realism is a superior philosophical position on the scientific method than Hypothetico-Deductivism, and following Haig (2014), calls for wider recognition of other important elements of scientific reasoning, such as abductive reasoning. Hubbard carefully details his proposed solution in *Corrupt Research*, but one does not have to accept Significant Sameness to accept the depth and power of this book. Given that many have called for more philosophy of science training in wake of the “reproducibility crisis,” Hubbard's *Corrupt Research* is a timely offering.

## Author contributions

The author confirms being the sole contributor of this work and approved it for publication.

### Conflict of interest statement

The author declares that the research was conducted in the absence of any commercial or financial relationships that could be construed as a potential conflict of interest.
